# Havruta-structured dialogue as a social–emotional competence intervention: multi-stakeholder evidence on university students’ mental health

**DOI:** 10.3389/fpsyg.2026.1831783

**Published:** 2026-05-28

**Authors:** Lingyun Deng, Fengni Yang, Binghao Guo, Ling Luo

**Affiliations:** 1 College of Education, Kookmin University, Seoul, Republic of Korea; 2 Guangzhou City Construction College, Guangzhou, China

**Keywords:** Havruta dialogue, mental health, social–emotional competence, university students, psychological wellbeing, structured dialogue

## Abstract

**Introduction:**

This study evaluated the preliminary efficacy of a Havruta-structured dialogue program in enhancing university students’ social–emotional competence (SEC) and mental health.

**Methods:**

Ninety-five students were randomly assigned to either a Havruta intervention group (*n* = 47) or an active control group (*n* = 48). Assessments were conducted at baseline (T0), post-intervention (T1), and follow-up (T2).

**Results:**

Compared with the control group, participants in the Havruta group showed significantly greater reductions in depressive symptoms, anxiety, and perceived stress, along with significantly greater improvements in psychological wellbeing and SEC, with benefits maintained through follow-up. Reliable change analysis further indicated a significantly higher proportion of clinically meaningful responders in the intervention group. Exploratory mediation analyses provided preliminary evidence consistent with SEC gains as one plausible intermediary associated with improved mental health outcomes. Exploratory trajectory analyses suggested heterogeneous response patterns, with baseline distress, loneliness, and dialogue-process quality emerging as candidate correlates of differential improvement. Triangulated data from multiple stakeholders further supported the intervention’s feasibility, engagement, and perceived process features within the present university context.

**Discussion:**

Overall, these findings suggest promising preliminary effects in a modestly sized university sample; however, larger fully powered randomized trials are needed to confirm the exploratory secondary findings, establish generalizability, and determine whether broader implementation is warranted.

## 1 Introduction

Mental health challenges among university students have become increasingly prevalent globally (Auerbach et al., 2018; Ibrahim et al., 2013; Song et al., 2023). Symptoms of depression, anxiety, and academic burnout, often exacerbated by prolonged academic pressure, are particularly common. These mental health difficulties not only impair students’ personal wellbeing but also negatively impact their academic performance and social functioning (Lötjönen et al., 2025; Richardson et al., 2012; Velert-Jiménez et al., 2025). Although traditional clinical interventions are effective, their campus-wide implementation is frequently hindered by mental health stigma and limited professional resources. Therefore, there is an urgent need to develop low-stigma, non-clinical group interventions that can be seamlessly integrated into routine university activities (Wang et al., 2023). Such approaches aim to embed mental health support within students’ daily interpersonal interactions, thereby offering preventive care and fostering holistic development.

Social–emotional competence (SEC)—comprising malleable skills such as self-awareness, emotion regulation, and interpersonal communication—serves as a crucial protective factor for university students’ mental health (Durlak et al., 2011; Grégoire et al., 2024; Taylor et al., 2017). Enhancing SEC builds psychological resilience, enabling students to foster stronger social support networks and cultivate a sense of belonging. Individuals possessing elevated SEC are significantly more predisposed to deploy adaptive emotion regulation strategies, notably cognitive reappraisal, to effectively mitigate negative affective states and sustain overall wellbeing (Aldao et al., 2010; Troy et al., 2010; Webb et al., 2012). Targeting the SEC through preventive interventions equips students with the essential skills to navigate current academic distress and future professional challenges (Reyes-Portillo et al., 2025).

Havruta represents a structured, dyadic dialogue format deeply rooted in foundational sociocognitive and dialogic learning theories (Asterhan and Schwarz, 2016; Howe et al., 2019; Mercer, 2013). Its core mechanism involves constructing a deep cognitive space through mutual questioning, in-depth inquiry, and perspective-taking (Eom and Lee, 2020; Schillings et al., 2021). Unlike casual social conversations, Havruta requires participants to actively coordinate their views and co-construct meaning within a structured, logical framework. Through repeated cycles of interpreting, questioning, and integrating information, students engage in rigorous academic discussion and the practice of advanced social interaction skills. This structured interaction mitigates individual isolation and fosters a sense of campus connectedness, thereby providing a viable and non-stigmatizing platform for mental health promotion (Welz et al., 2023).

Evaluating psychological interventions solely through self-report inventories introduces susceptibility to common method variance, constraining the comprehensiveness of clinical evaluations (Amaro et al., 2025; Conway and Lance, 2010; Spector, 2006). To address this inherent limitation, this study incorporated a multi-stakeholder assessment framework. Within the context of this study, “stakeholders” explicitly refer to the triad of individuals directly involved in the intervention process: the focal participating students, their randomly assigned peer dialogue partners (i.e., fellow university students enrolled in the same intervention cohort), and the trained session facilitators. This framework was designed to gather supplementary process and engagement data from these dialogue partners and facilitators. Although these proximal, non-blinded observational metrics cannot eliminate common method bias or serve as independent outcome validations, triangulating these sources provides crucial contextual evidence regarding intervention feasibility, implementation fidelity, and participant engagement (Pointon-Haas et al., 2023). Specifically, this study examined the following questions and expectations:

1. Participants in the Havruta-structured dialogue intervention were expected to demonstrate greater improvements in mental health outcomes and SEC than those in an active control group.
2. Gains in SEC were examined as a plausible intermediary process that might be associated with improved mental health outcomes.
3. Baseline psychological distress, loneliness, and dialogue-process quality were explored as candidate correlates of differential trajectories of improvement.

## 2 Methods

### 2.1 Design overview and assessment schedule

**Figure 1:**
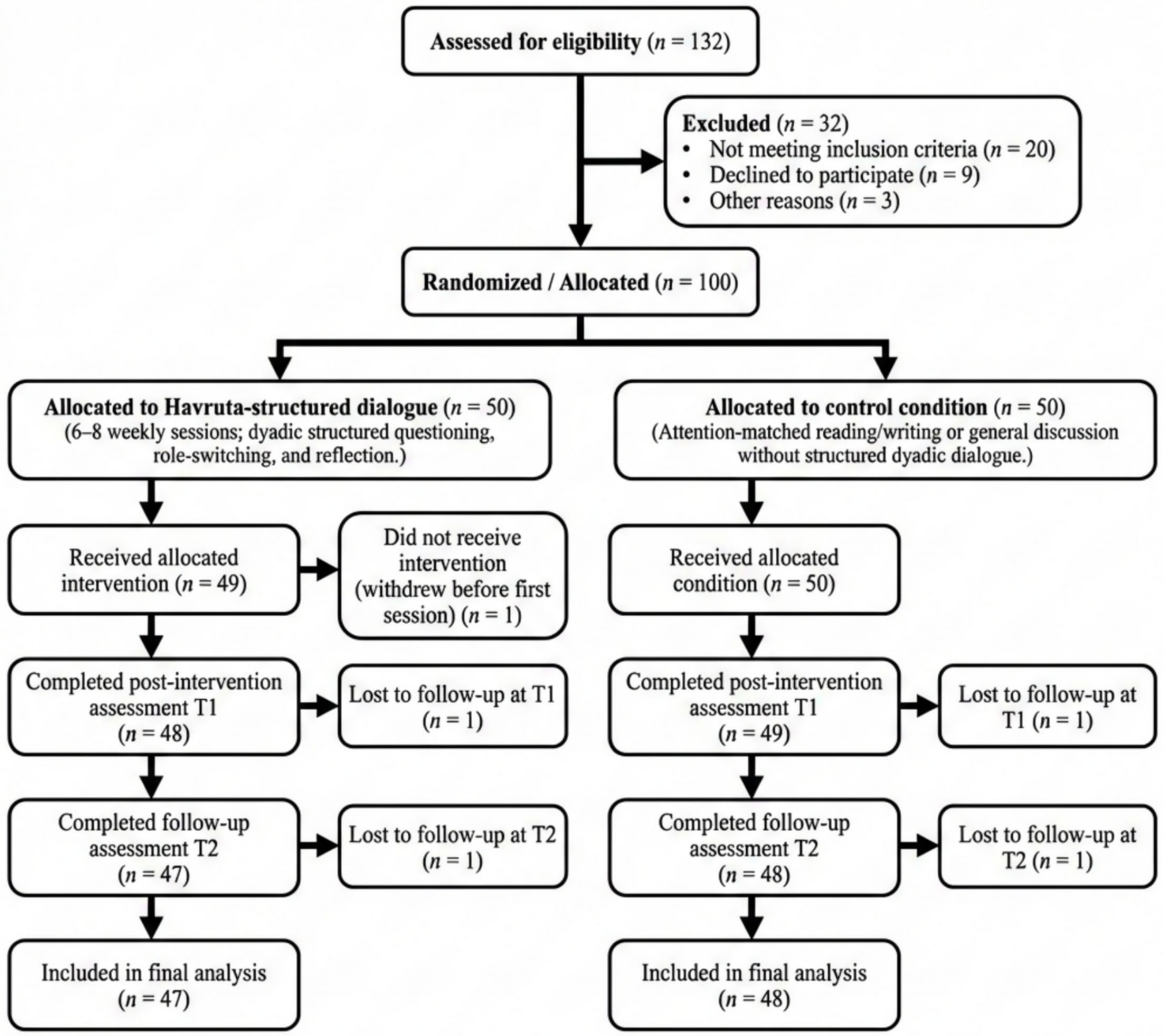
Raincloud plots of pre-post-follow-up changes in social–emotional competence and mental health outcomes by group: **(A)** PHQ-9 depressive symptoms, **(B)** GAD-7 anxiety symptoms, **(C)** PSS-10 perceived stress, and **(D)** SEC total.

This study used a longitudinal experimental design with two groups to investigate the effects and mechanisms of the Havruta-structured dialogue intervention on the SEC and on college students’ mental health outcomes. The study was integrated into a university-wide mental health and personal development elective program, ensuring access to a diverse student population. The intervention group received Havruta-structured dyadic dialogue training, whereas the control group engaged in attention-matched psychoeducational activities, including independent reading and unstructured group discussions on university adaptation. All sessions across both conditions were administered by four graduate research assistants who held bachelor’s degrees in psychology and had completed a standardized 16-h training program covering group facilitation and crisis management protocols. Participants were randomized at the individual level using a computer-generated random number sequence created by an independent researcher unaffiliated with data collection. Allocation concealment was maintained utilizing sequentially numbered, opaque, sealed envelopes. Given the interactive nature of the intervention, participants and facilitators could not be blinded; however, outcome assessors and data analysts remained strictly blinded to group assignments throughout the trial. The overall pathway and sample flow are presented in Figure 1.

Three measurement time points were established to capture immediate post-intervention changes and evaluate subsequent maintenance effects: T0 (baseline), which was conducted following group allocation but before the intervention; T1 (post-intervention), which was carried out right after the intervention to measure the short-term effect; T2 (follow-up), which was conducted exactly 6 weeks post-intervention to evaluate the persistence of the observed effects (Madrid-Cagigal et al., 2025). This three-point longitudinal design facilitates trajectory modeling of mental health outcomes and proximal SEC indicators, thereby establishing the temporal precedence required for testing mediating mechanisms.

In terms of sample composition, this study eventually included two sets of data for primary analysis: the intervention group (*n* = 47) and the control group (*n* = 48). During the intervention implementation and follow-up phases, compliance and implementation quality information (e.g., attendance, conversation completion rate, number of dialogue rounds, and key conversational behaviors) were simultaneously recorded for subsequent dose–response relationship and heterogeneity analysis. To reduce common method bias arising from a single self-assessment, this study introduced multi-source evidence (including evaluations from the participant’s assigned peer partner, observational ratings from trained facilitators, and structured process documentation) into the measurement process. By triangulating these multi-source metrics with concurrent student self-assessments, a robust evidence chain for both outcome evaluation and mechanistic interpretation was established.

### 2.2 Participants

Participants were university students recruited through a combination of convenience sampling and targeted campus project announcements. Recruitment channels included campus course announcements, college notices, and posts by counselors and class groups, covering students of different grades and academic backgrounds. All participants who agreed to participate in this study provided informed consent and were clearly informed of the purpose of the research, the procedures for participation, the possible risks and benefits, the principle of protecting personal information, and their right to withdraw at any time. All procedures strictly adhered to ethical guidelines for psychological research, incorporating specific protocols for risk identification and crisis management. For individuals identified during screening as having major psychological crises or self-harm tendencies, the research team did not initiate the intervention procedures. However, it activated the previously planned referral and assistance channels and recommended that they contact the campus psychological counseling center or relevant professional services to ensure the participants’ safety.

Inclusion criteria were as follows: (1) aged 18 years or older and currently enrolled as a full-time university student; (2) possessing the cognitive and linguistic capacity to comprehend the study protocol and complete the baseline assessments; (3) expressing willingness to participate in dyadic structured dialogue activities; and (4) committing to attend follow-up assessments at T1 and T2. Exclusion criteria encompassed: (1) individuals experiencing an acute psychological crisis or presenting significant self-harm risks necessitating immediate clinical intervention; (2) those currently suffering from severe mental disorders incompatible with interactive group tasks; (3) participants concurrently engaged in intensive psychotherapy that could confound study outcomes; and (4) individuals unable to complete core procedures due to logistical or cognitive limitations.

To improve sample homogeneity and control for potential confounding factors, demographic and background information for participants, such as sex, grade, major, history of psychological consultation, and major life events in recent days, was collected during the baseline period. These data were used to control for potential confounding factors in the following models or explored as stratification/moderation variables. Given that the intervention depended on peer interactions, the details of the implementation situation (such as pairing methods, pairing stability, and participants’ willingness to be paired) were also recorded during the process, and this enabled the feasibility of the intervention and the consistency of its implementation to be assessed.

The primary analysis adhered to the intention-to-treat principle, ensuring that all 95 randomized participants were retained in their originally assigned groups regardless of intervention adherence or assessment completion (McCoy, 2017). Rather than relying on *ad hoc* imputation or completer-only subsets, linear mixed-effects models utilizing restricted maximum likelihood estimation were employed. This approach provides unbiased parameter estimates under the assumption that data are missing at random, thereby preserving the structural integrity of the initial randomization despite selective attrition. To enhance the credibility of the interpretation results, multi-source evidence was considered within the feasible range (peers, teachers/counselors, process records), and together with participants’ self-report data, a comprehensive evaluation of the intervention’s effectiveness and its underlying mechanisms was constructed.

### 2.3 Intervention description

**Figure 2:**
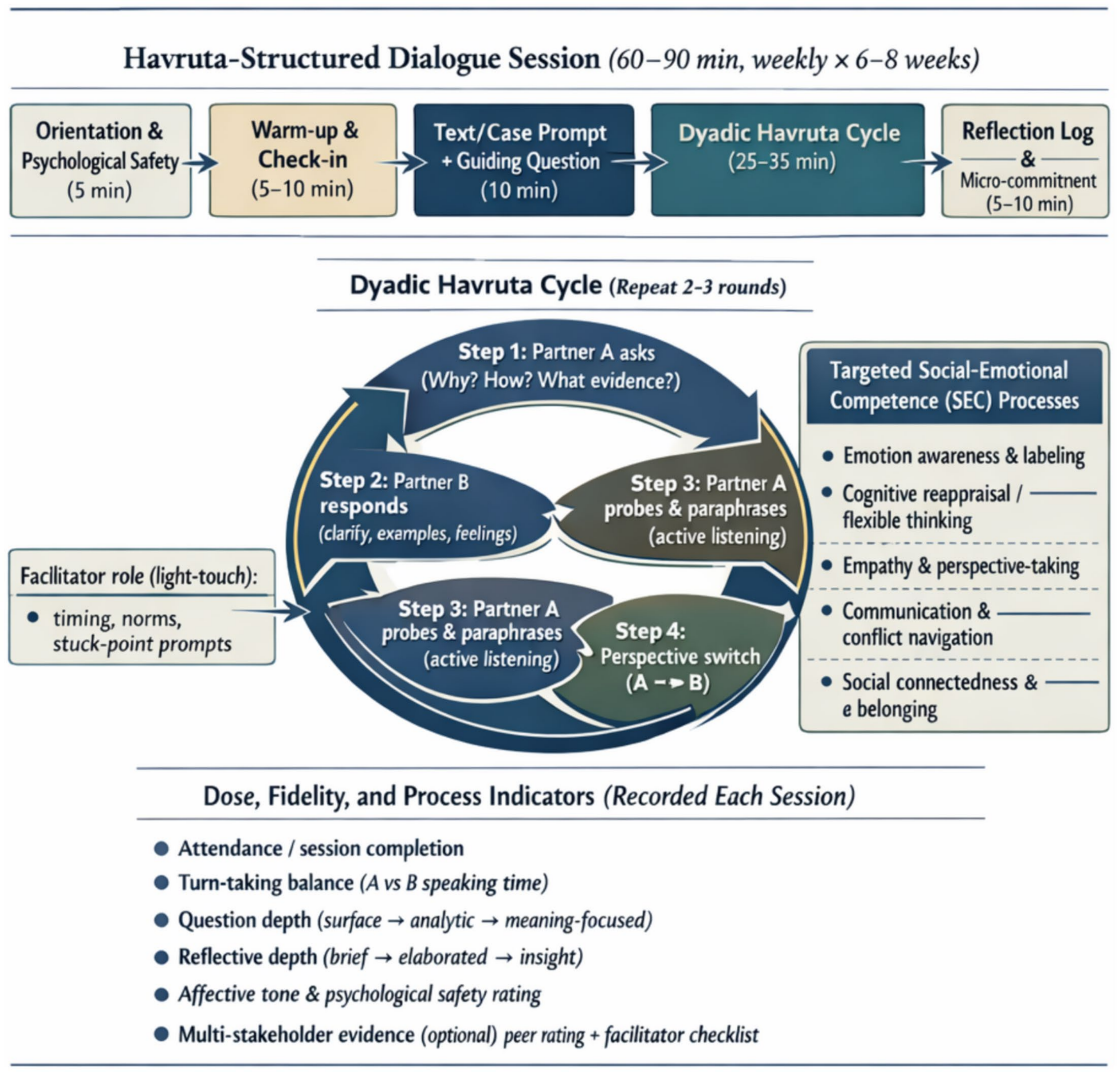
Structure and components of the Havruta-structured dialogue sessions.

This study used Havruta-structured dialogue as the primary intervention, aiming to enhance university students’ SEC and subsequently promote mental health through a standardized dyadic reciprocal process. The intervention design prioritized structural clarity, replicability, and quantifiable processes, with the core components illustrated in Figure 2.

The intervention consisted of six to eight weekly sessions, each lasting 60–90 min. A standard session progressed sequentially through establishing psychological safety norms (e.g., respectful listening and confidentiality), emotional check-ins, collaborative reading of case prompts with guiding questions, the core dyadic Havruta cycle, and a concluding joint reflection. The core dyadic cycle consisted of sequential inquiry, response, active paraphrasing, role reversal, and joint meaning construction. This cycle was typically repeated two to three times per session to foster reciprocal communication and deepen cognitive engagement. Facilitators utilized a non-directive moderation strategy—focusing on time management, norm reinforcement, and structural cuing—to ensure that interactions remained peer-driven (i.e., conducted exclusively between the paired student participants) rather than didactic.

To maintain implementation fidelity, facilitators adhered to session checklists, and an independent rater evaluated a random 20% sample of audio-recorded sessions using a standardized fidelity rubric. Concurrently, this study continuously recorded process quality indicators, including attendance, reciprocity of speech, depth of questioning, and psychological safety scores. These data were combined with peer evaluation/facilitator verification forms to form process evidence, which was used in the following analyses of dose–response, heterogeneity, and underlying mechanisms.

### 2.4 Control condition and implementation fidelity

This study created an equal-attention control condition to control for non-specific factors such as time investment, focus of attention, and interaction with peers. The active control condition was strictly attention-matched, consisting of eight weekly 90-min sessions supervised by the same trained facilitators to govern non-specific factors such as time investment and group format. Participants engaged in structured reading and independent written reflections utilizing the identical university adaptation and mental health materials provided to the intervention group. Following individual reflections, facilitators guided unstructured, open-group discussions. Crucially, the control sessions explicitly excluded the structured dyadic reciprocal mechanisms—specifically, mutual questioning, active paraphrasing, and role reversal—ensuring that between-group differences effectively isolated the unique impact of the Havruta dialogic structure. This matched design ensured that any observed between-group differences specifically reflected the incremental therapeutic effect of the structured dialogue mechanism (Alvarez et al., 2025).

To reduce the impact of contamination and bias, two groups of activities were organized at different times/in different classes to the best of the researchers’ abilities, and it was explicitly stipulated that there should be no cross-group dissemination of materials. Whether participants received other psychological interventions or systematic counseling during the study period was recorded, and covariates were included or sensitivity analyses were performed when necessary.

Consistency was assessed through three categories of indicators: dosage (attendance rate, completion rate, duration of participation), adherence (whether key steps were carried out according to the protocol and whether structured dialogue components were introduced into the control group), and quality (reciprocity in the intervention group, depth of questioning, restatement confirmation, completion rate of reflection, psychological safety score). Standardized checklists were used to record deviations and their causes, and explanations were given in the results interpretation to enhance the robustness and reproducibility of the conclusions.

### 2.5 Measures

The measurement framework of this study comprised two main components: mental health outcomes and SEC, and a repeated-measures design with three time points was adopted: T0 (baseline), T1 (post-intervention), and T2 (follow-up, conducted 6 weeks post-intervention).

Mental health outcomes and SEC were assessed at all time points using rigorously validated Korean and Chinese translations of standardized psychometric instruments. To assure linguistic equivalence and cultural appropriateness, the research team strictly avoided *ad hoc* or direct translations. Instead, we exclusively administered the widely recognized local-language versions of these scales that have been previously adapted, psychometrically validated, and extensively used in relevant clinical and educational research within this specific cultural context. The central mechanistic construct, SEC, was rigorously operationalized using the Social–Emotional Competence Questionnaire. This specific inventory features a well-established dimensional structure encompassing five distinct facets: self-awareness, social awareness, self-management, relationship management, and responsible decision-making. To optimize manuscript conciseness while ensuring methodological transparency, comprehensive technical details regarding all utilized scales—including the Patient Health Questionnaire-9, Generalized Anxiety Disorder-7, Perceived Stress Scale-10, WHO-5 Well-Being Index, and the aforementioned SEC measure—are synthesized in Table 1. This table explicitly delineates their target constructs, item counts, scoring formats, structural validation references, and the specific internal consistency coefficients observed within the current analytic sample.

**Table 1:** Summary of measurement instruments for mental health and social–emotional competence.

Instrument	Target constructs/facets	Items	Response scale	Reliability (*α*)	Reference
PHQ-9	Depressive symptoms	9	0 (not at all) to 3 (nearly every day)	0.89	Kroenke, Spitzer, and Williams (Kroenke et al., 2001)
GAD-7	Anxiety symptoms	7	0 (not at all) to 3 (nearly every day)	0.92	Spitzer, Kroenke, Williams, and Löwe (Spitzer et al., 2006)
PSS-10	Perceived stress	10	0 (never) to 4 (very often)	0.86	Cohen, Kamarck, and Mermelstein (Cohen et al., 1983)
WHO-5	Psychological wellbeing	5	0 (at no time) to 5 (all of the time)	0.88	Topp, Østergaard, Søndergaard, and Bech (Topp et al., 2015)
SECQ	SEC facets (emotion awareness, regulation, empathy, conflict navigation, social connectedness)	25	1 (strongly disagree) to 5 (strongly agree)	0.91	Tan, Wang, Xiao, Jing, Chen, and Yao (Tan et al., 2025); Oh, Shin, and Choi (Oh et al., 2022)

**Figure 3:**
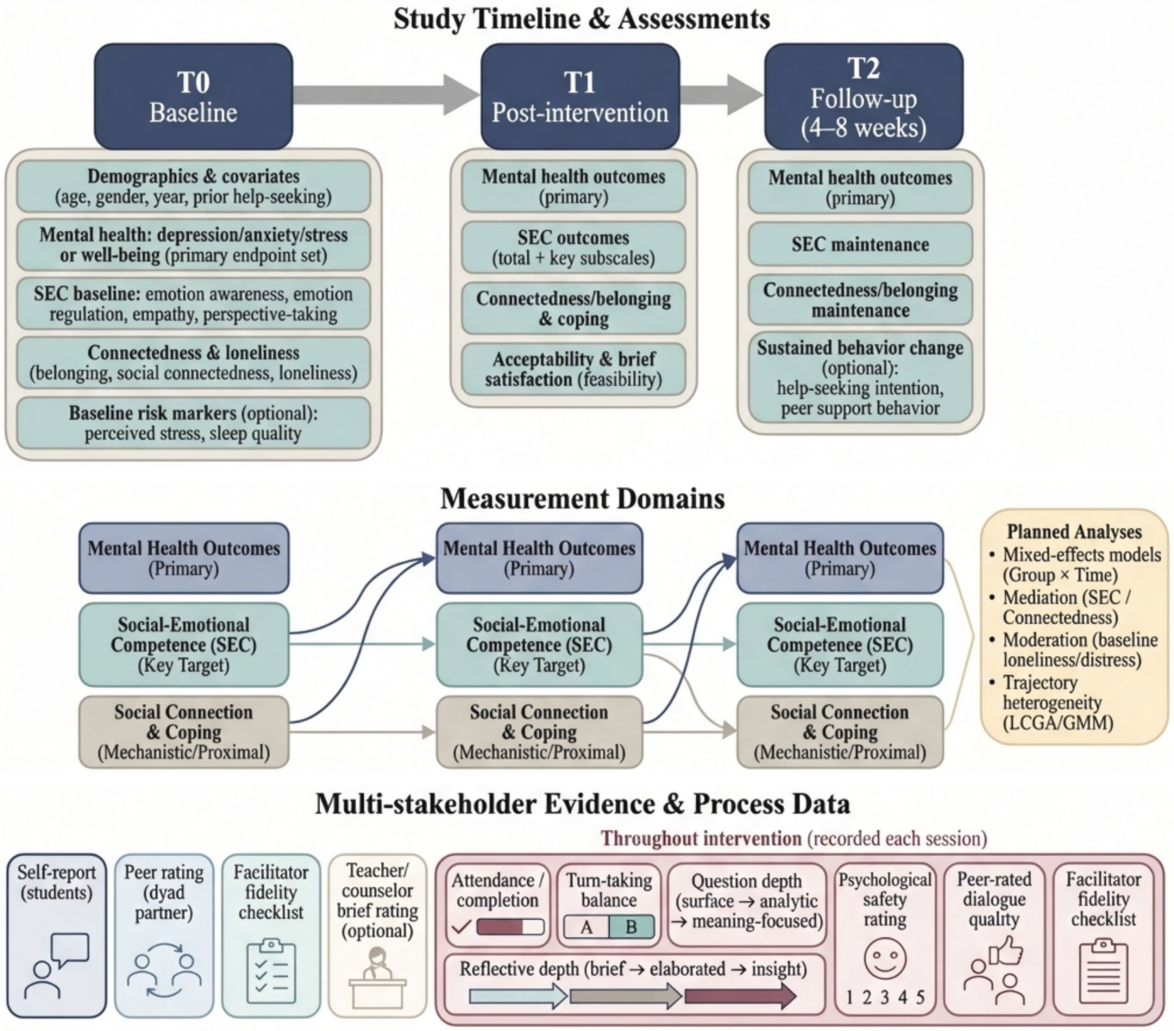
Measurement framework and assessment timeline for social–emotional competencies and mental health outcomes (T0–T2) plus multi-stakeholder evidence collection.

To enhance interpretability and reduce common method bias, this study added multi-stakeholder evidence and process indicators in addition to self-assessment, such as peer assessment, implementation consistency checked by facilitators/research assistants (e.g., execution of key steps), and brief observational assessment by teachers/counselors as needed. Meanwhile, intervention process indicators (such as attendance and completion rate, reciprocal dialogue, depth of questioning, depth of reflection, and psychological safety score) were recorded for the analysis of dose–response relationships, explanation of effect heterogeneity, and as additional evidence to explain the mechanism. The location and purpose of the relevant data in this study timeline are integrated and presented in Figure 3.

### 2.6 Data analytic strategy

The data analysis in this study followed predetermined protocols for reproducibility, using two-tailed tests and reporting effect sizes and 95% confidence intervals. First, descriptive statistics were conducted to describe the demographic characteristics and baseline scale distribution, assess comparability between groups at T0, and analyze the pattern of attrition and missing data to assess the presence of selection bias. Baseline statistical testing provided the quantitative foundation necessary for all subsequent longitudinal modeling.

To mitigate the potential inflation of Type I error associated with multiple testing, changes in depressive symptoms, anxiety, and overall SEC were designated *a priori* as the primary confirmatory endpoints. Longitudinal trajectories and between-group differences for these primary variables were estimated utilizing linear mixed models. This robust analytic framework is optimally suited for repeated-measures designs that may involve subject attrition (Boot et al., 2013; Curran et al., 2010; Kinser and Robins, 2013). Under the missing-at-random assumption, the restricted maximum likelihood estimation inherent in these models mathematically accounts for missingness without requiring raw-data transformation. All subsequent evaluations, including moderation, mediation, and trajectory classifications, were strictly defined as exploratory secondary analyses. For these exploratory models, nominal probability values were reported without formal alpha adjustment and should therefore be interpreted with appropriate analytical caution. Furthermore, sensitivity analyses were conducted to assess the robustness of these estimates to potential deviations from the missing-at-random assumption. Consequently, the change scores reported in the results represent estimated marginal means adjusted for covariates, rather than simple arithmetic differences between raw scores.

To assess clinical significance beyond arbitrary statistical thresholds, the Reliable Change Index (RCI) was calculated to account for instrument-specific measurement error (Enders, 2001; Graham, 2009; Ibrahim and Molenberghs, 2009). Participants were classified as demonstrating reliable improvement, no change, or reliable deterioration based on whether their score changes exceeded the instrument-specific measurement error margin. Risk differences (RDs) and risk ratios (RRs) were subsequently computed to quantify the absolute and relative probability of improvement.

Mechanism testing evaluated the mediating role of SEC using bootstrap-based indirect-effect estimation, an approach that maximizes statistical power while relaxing normal distribution assumptions (Hayes, 2009; Preacher and Hayes, 2008; Shrout and Bolger, 2002). Furthermore, hierarchical linear regression was performed in three progressive blocks. This approach deliberately included demographic covariates in the initial models to confirm their non-significant confounding effects before introducing focal predictors, thereby isolating the incremental predictive validity of intervention-related SEC gains.

Given the constraints of the current sample size relative to the analytical scope, latent class growth analysis and subsequent predictive modeling were strictly designated as exploratory secondary analyses. To capture unobserved population heterogeneity, this exploratory application of latent class growth analysis was conducted on longitudinal symptom scores, determining optimal model fit and objective classification of discrete trajectory patterns via the Bayesian Information Criterion and entropy metrics (Nylund-Gibson and Choi, 2018). Consequently, the multivariable logistic regression model used to predict follow-up improvement probabilities, which is visualized as an exploratory nomogram, should be interpreted as a preliminary hypothesis-generating framework rather than a definitive clinical tool.

Finally, cross-domain coupling network analysis was conducted using a Gaussian Graphical Model based on regularized partial correlations (Epskamp et al., 2018). This approach mapped the complex network architecture among standardized conversational process metrics, multidimensional SEC facets, and specific mental health symptoms. This methodology ensures analytical validity by strictly utilizing quantitative, structured assessment scores rather than subjective free-text evaluations.

## 3 Results

### 3.1 Main impacts on mental health result from T0–T2

**Figure 4:**
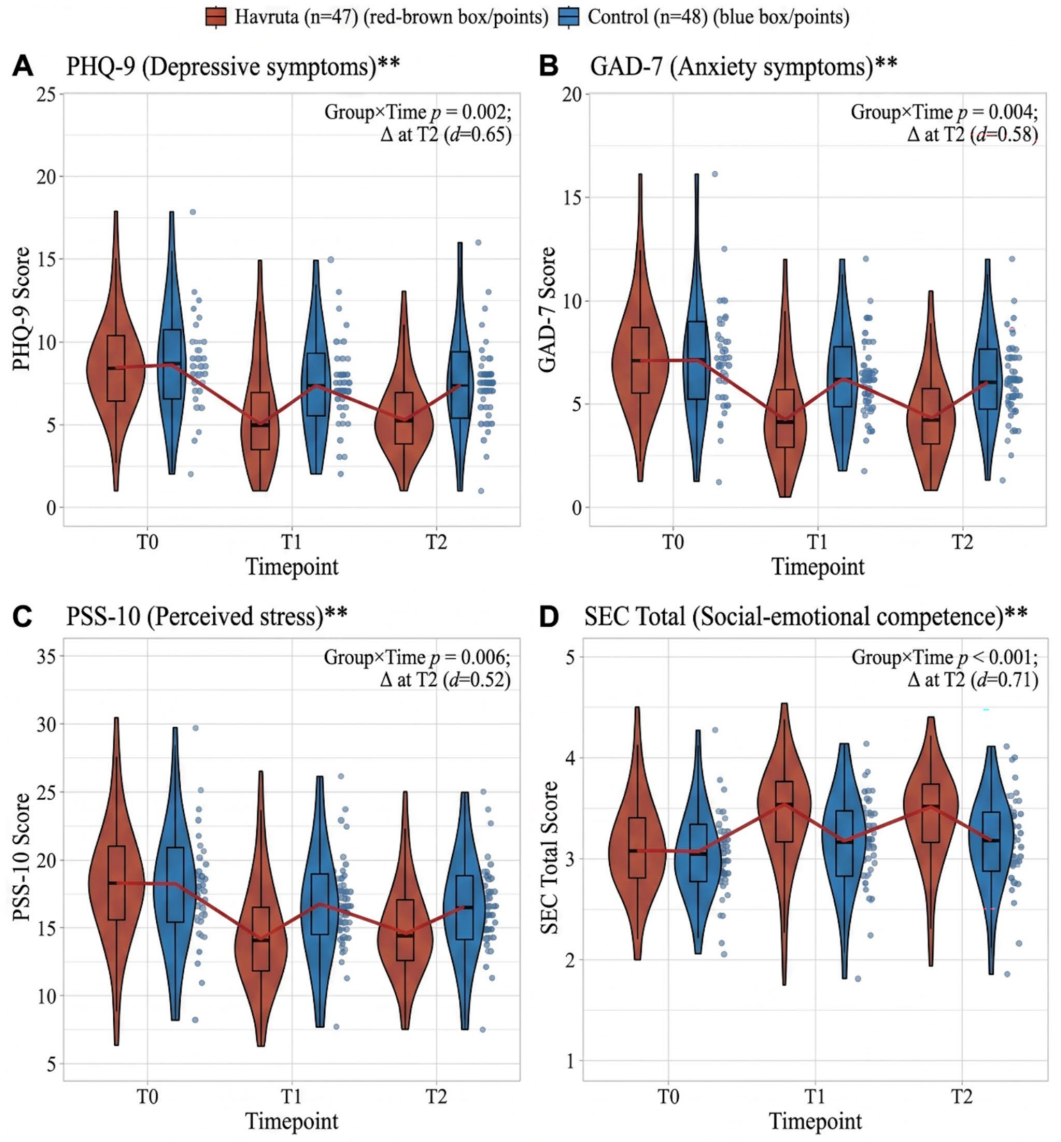
Raincloud plots of pre-post-follow-up changes in social–emotional competence and mental health outcomes by group.

Primary analyses evaluated the overarching effects of the Havruta-structured dialogue intervention on mental health and SEC trajectories from T0 to T2. The two groups were comparable at baseline in terms of mental health and overall SEC, with no significant baseline differences. Longitudinal modeling revealed that the intervention group showed more pronounced improvement trajectories across multiple mental health scales. These enhancements were evident immediately after the intervention and sustained throughout the follow-up period. This suggests that the intervention effects not only have a short-term effect but also have a significant maintenance effect. Notably, the specific data distributions depicted within the raincloud diagrams (Figure 4)—particularly the occasional observation of jittered points outside the density plot boundaries for controls—are attributable to the selected horizontal width parameter for data jittering combined with distinct underlying data concentrations and variability between groups after intervention.

Regarding specific symptomatology, the intervention group exhibited significantly steeper trajectories of symptom reduction for both depression and anxiety from T0 to T1, maintaining these lower levels through T2. Similarly, perceived stress declined markedly in the intervention condition, whereas it remained relatively stable in the control group. Conversely, psychological wellbeing indicators demonstrated a more prominent upward trajectory in the intervention group, suggesting that this dialogic intervention not only mitigated negative symptomatology but also facilitated the accumulation of positive psychological resources. The differences between these groups over time were statistically significant. These longitudinal shifts are presented in Table 2 as estimated marginal means and adjusted mean differences, with the modeled covariates accounted for.

**Table 2:** Between-group effects of Havruta-structured dialogue on social–emotional competence and mental health outcomes by time points.

Outcome	T0 baseline Havruta	T0 baseline control	T1 post Havruta	T1 post control	T2 follow-up Havruta	T2 follow-up control	Between-group diff. at T1 (Adj. MD, 95% CI)	*p*-value	Between-group diff. at T2 (Adj. MD, 95% CI)	*p*-value	Std. effect (d) at T2
Depressive symptoms (PHQ-9)	8.63 (4.12)	8.51 (4.06)	5.14 (3.62)	7.41 (4.08)	5.42 (3.78)	7.28 (4.21)	−2.21 (−3.62, −0.81)	0.002	−2.02 (−3.48, −0.56)	0.008	0.49
Anxiety symptoms (GAD-7)	7.32 (3.51)	7.18 (3.44)	4.61 (3.06)	6.45 (3.39)	4.88 (3.14)	6.26 (3.52)	−1.72 (−2.89, −0.55)	0.004	−1.41 (−2.61, −0.21)	0.022	0.41
Perceived stress (PSS-10)	18.47 (5.26)	18.29 (5.18)	14.23 (4.97)	16.98 (5.12)	14.61 (5.08)	16.62 (5.35)	−2.58 (−4.42, −0.74)	0.006	−2.12 (−4.02, −0.22)	0.029	0.39
Psychological wellbeing (WHO-5)	48.72 (16.31)	49.10 (15.86)	59.84 (15.02)	52.36 (15.41)	58.93 (15.44)	52.91 (15.68)	+8.34 (+2.11, +14.58)	0.009	+6.52 (+0.21, +12.83)	0.043	0.39
Social–emotional competence	3.12 (0.47)	3.11 (0.45)	3.58 (0.44)	3.23 (0.46)	3.54 (0.46)	3.27 (0.45)	+0.34 (+0.17, +0.51)	<0.001	+0.28 (+0.10, +0.46)	0.003	0.61

Data are presented as mean (standard deviation) unless otherwise specified. Adjusted mean differences (Adj. MD) represent the estimated marginal means between groups derived from linear mixed-effects models that account for baseline covariates. Consequently, the Adj. MD may not exactly match the arithmetic differences of the raw, unadjusted means presented in the descriptive columns.

In line with the results for mental health, SEC, as the core intervention target, showed greater SEC in the intervention group than in the control group at all time points, indicating that the intervention was effective in reaching the specified proximal competence objectives. Figure 4 provides a visual representation of the overall distribution shifts for both groups across the three time points (T0, T1, and T2) utilizing raincloud plots. The intervention group showed a shift toward lower scores on symptom-related indicators and higher scores on SEC, with individual-level variability among responders and non-responders. These individual-level variances provide a critical empirical foundation for the subsequent heterogeneity and mechanistic analyses.

### 3.2 Reliable and meaningful change

In addition to the mean difference and statistical significance results, this study further examined the clinical significance of the intervention at the individual level by using the RCI to classify changes from T0 to T1. The participants were categorized into “reliable improvement,” “no significant change,” or “reliable deterioration.” This analysis can directly answer whether the intervention brought about practical benefits to the individual that “exceeded measurement error,” which improves the interpretability and practical value of the results.

The results showed that in terms of symptomatic indicators such as depression, anxiety, and perceived stress, the intervention group had a higher proportion of “reliable improvement” overall than the control group, and the proportion of “reliable deterioration” was relatively lower. Regarding psychological wellbeing indicators, the proportion of reliable improvements in the intervention group was also higher. The difference in the probability of reliable improvement across the two groups was in the same direction. Hence, it can be concluded that Havruta-structured dialogue not only offers overall benefits but also results in a higher proportion of people showing meaningful improvement in their scores.

According to the distribution pattern, the control group is mostly in the “no significant change” category, indicating that routine activities provided only a small short-term benefit for mental health when participants received equal attention. In contrast, the improvement rate of the intervention group was more obvious, indicating that the structured dialogue-based social–emotional training was more beneficial for relieving symptoms in the short term and enhancing the wellbeing of students with a certain degree of depression. The results of this RCI classification, alongside the RDs and relative RRs evaluating the absolute and relative probabilities of improvement across groups, are detailed in Table 3. All proportional differences yielded statistically significant chi-square or Fisher’s exact test results at the 0.05 level.

**Table 3:** Clinically meaningful change in mental health outcomes: Reliable Change Index (RCI) and responder classifications from pre- to post-intervention.

Outcome (T0 → T1)	Group	Improved, *n* (%)	Unchanged, *n* (%)	Deteriorated, *n* (%)	Risk difference (improved), % (95% CI)	RR (improved) (95% CI)	p (χ^2^/Fisher’s exact)
PHQ-9 (depressive symptoms)	Havruta	19 (40.4)	25 (53.2)	3 (6.4)	+21.6 (+4.1, +38.7)	2.15 (1.09, 4.26)	0.018
	Control	9 (18.8)	34 (70.8)	5 (10.4)			
GAD-7 (anxiety symptoms)	Havruta	17 (36.2)	27 (57.4)	3 (6.4)	+19.5 (+2.6, +36.2)	2.17 (1.03, 4.58)	0.033
	Control	8 (16.7)	35 (72.9)	5 (10.4)			
PSS-10 (perceived stress)	Havruta	15 (31.9)	29 (61.7)	3 (6.4)	+17.3 (+1.0, +33.3)	2.18 (0.99, 4.79)	0.049
	Control	7 (14.6)	36 (75.0)	5 (10.4)			
WHO-5 (wellbeing; higher is better)	Havruta	18 (38.3)	26 (55.3)	3 (6.4)	+17.5 (+0.7, +34.0)	1.84 (1.00, 3.40)	0.046
	Control	10 (20.8)	33 (68.8)	5 (10.4)			

RCI = Reliable Change Index; RR = relative risk; CI = confidence interval. Participants were classified based on whether their score changes exceeded the instrument-specific measurement error margin. Risk difference indicates the absolute percentage increase in the reliable improvement probability in the Havruta group relative to the control group. RR indicates the relative probability multiplier for achieving improvement.

### 3.3 Exploratory moderators and candidate predictors

To further examine possible sources of individual differences in intervention response, this study conducted exploratory, predictive, and moderation analyses building on the primary outcome findings. Two questions were considered. First, the analyses examined whether changes in SEC and social connectedness during the intervention were associated with follow-up mental health status. Second, they explored whether selected baseline characteristics were associated with differential patterns of benefit following the intervention.

In the exploratory predictive model, hierarchical regression was conducted with depressive symptoms at follow-up as the representative outcome. The initial model incorporated baseline symptoms and demographic covariates. Following the trial protocol, demographic variables including sex, year in university, and prior help-seeking were retained across subsequent blocks regardless of their individual statistical significance. This conservative approach was intended to control for *a priori* theoretical confounders while examining the additional explanatory contribution of intervention-related changes in SEC. The results indicated that baseline symptom levels remained strongly associated with follow-up symptom status. In addition, greater increases in SEC and greater improvement in social connectedness during the intervention period were associated with lower follow-up symptom levels, contributing additional explained variance to the model. Notably, after changes in SEC and social connectedness were introduced, the main effect of the group was attenuated. This pattern is broadly consistent with the possibility that changes in these proximal factors may be relevant to later mental health outcomes, although the analyses are exploratory and should not be interpreted as confirming an indirect mechanism. The parameter estimates and model fit statistics of the hierarchical model are presented in Table 4.

**Table 4:** Hierarchical regression predicting follow-up mental health from baseline covariates and intervention-related changes in social–emotional competence.

Predictor	Model 1 B (SE)	β	*p*	Model 2 B (SE)	β	*p*	Model 3 B (SE)	β	*p*
Intercept	7.06 (0.41)		<0.001	7.01 (0.40)		<0.001	7.00 (0.39)	—	<0.001
group (Havruta *vs*. control)	−0.88 (0.54)	−0.16	0.106	−0.66 (0.52)	−0.12	0.21	−0.42 (0.51)	−0.08	0.409
PHQ-9 at T0	0.56 (0.07)	0.63	<0.001	0.49 (0.07)	0.56	<0.001	0.46 (0.07)	0.52	<0.001
Sex (female = 1)	0.38 (0.47)	0.07	0.42	0.29 (0.45)	0.05	0.52	0.22 (0.44)	0.04	0.616
Year in university	−0.21 (0.18)	−0.10	0.243	−0.18 (0.17)	−0.09	0.292	−0.16 (0.17)	−0.08	0.343
Prior help-seeking (Yes = 1)	0.64 (0.51)	0.11	0.213	0.51 (0.49)	0.09	0.303	0.46 (0.48)	0.08	0.344
Δ SEC total (T1 − T0)				−1.18 (0.33)	−0.31	<0.001	−0.84 (0.34)	−0.22	0.016
Δ Social connectedness (T1 − T0)							−0.91 (0.29)	−0.26	0.002

Demographic covariates (sex, year in university, prior help-seeking) were strictly retained in Models 2 and 3 following a priori study protocols to control for theoretical confounders, regardless of their individual statistical significance in Model 1.

In the moderation analysis, this study examined the influence of initial levels and resource differences on the intervention effect through interaction terms in the longitudinal model. Longitudinal moderation analysis indicated that individuals presenting with higher initial distress levels experienced significantly greater symptom reductions following the intervention and throughout the follow-up period. Participants reporting higher baseline loneliness or fewer social resources demonstrated greater outcome gains, indicating that the Havruta intervention yields stronger compensatory benefits for individuals entering the program with constrained interpersonal networks and elevated emotional distress. Furthermore, prior psychological help-seeking significantly moderated the outcomes. This suggests that students with existing mental health literacy or prior motivation are more adept at translating structured dialogue training into sustained self-regulation and adaptive psychological practices. The estimated direction and significance of the moderating effects are shown in Table 5.

**Table 5:** Moderators of Havruta effects: baseline distress, loneliness, social connectedness, and prior help-seeking.

Moderator (baseline)	Interaction term tested	Estimate (SE)	t	*p*
Baseline distress (PHQ-9 T0)	Group × Time × Distress	−0.27 (0.09)	−3.02	0.003
Loneliness	Group × Time × Loneliness	−0.21 (0.10)	−2.10	0.038
Social connectedness	Group × Time × Connectedness	+0.19 (0.09)	2.07	0.041
Prior help-seeking (Yes/No)	Group × Time × Help-seeking	−0.73 (0.31)	−2.36	0.02

A negative interaction estimate indicates that higher baseline levels of the specific moderator (e.g., baseline distress or loneliness) are associated with a more pronounced reduction in symptom scores over time, signifying a stronger compensatory effect of the intervention for these individuals.

### 3.4 Mediation and mechanism tests

Exploratory mediation models were constructed to examine whether the observed intervention–outcome associations were statistically consistent with changes in SEC serving as a plausible intermediary process. Change in SEC during the intervention period was specified as the mediator, and mental health outcomes at follow-up were treated as the dependent variables. All models were adjusted for baseline outcome levels and key covariates, and indirect effects with 95% confidence intervals were estimated using bootstrapping. The unstandardized path coefficients (denoted as B , distinct from the standardized β reported in prior regression models), together with the total, direct, and indirect effects, are presented in Table 6. In bootstrap mediation analysis, statistical inference for indirect effects was based on whether the 95% confidence intervals excluded zero rather than on conventional *p*-values.

**Table 6:** Exploratory mediation analysis: changes in social–emotional competence as a plausible intermediary of intervention–outcome associations in mental health.

Outcome variable (at T2)	Path a: Group → ΔSEC, B (SE)	Path b: *Δ*SEC → Outcome, B (SE)	Direct effect (c′), B (SE)	Total effect (c), B (SE)	Indirect effect (a × b), bootstrap B (95% CI)	Mediation proportion (%)
Depressive symptoms (PHQ-9)	0.34 (0.07)**	−2.46 (0.72)**	−0.88 (0.61)	−1.72 (0.64)**	−0.84 (−1.42, −0.34)	48.9
Anxiety symptoms (GAD-7)	0.34 (0.07)**	−1.98 (0.61)**	−0.54 (0.50)	−1.21 (0.52)*	−0.67 (−1.16, −0.26)	55.4
Perceived stress (PSS-10)	0.34 (0.07)**	−3.11 (0.94)**	−0.92 (0.83)	−1.98 (0.86)*	−1.06 (−1.83, −0.41)	53.5
Psychological wellbeing (WHO-5)	0.34 (0.07)**	+8.72 (2.84)**	+3.07 (2.86)	+6.05 (2.93)*	+2.98 (+1.09, +5.31)	49.3

Path coefficients are unstandardized estimates (B), distinct from the standardized β reported in prior regression models. Path a represents the association between group assignment and change in SEC; Path b represents the association between SEC change and the outcome. Indirect effects were estimated using 5,000 bootstrap resamples and were interpreted based on whether the 95% confidence interval excluded zero. Given the exploratory nature of these analyses, the findings should be interpreted cautiously and should not be taken as confirmation of a causal mechanism. * *p* < 0.05, ** *p* < 0.01.

The results showed that participants in the intervention group demonstrated greater gains in SEC during the intervention period than those in the control group, indicating that the program was associated with improvement in this proximal target. In turn, greater improvement in SEC was associated with more favorable follow-up mental health outcomes, reflected in lower symptom levels and higher psychological wellbeing. Indirect effects were observed across multiple outcomes, yielding a pattern consistent with SEC functioning as one plausible intermediary pathway. However, these analyses remain exploratory and should not be interpreted as confirmation of the mechanism.

After the inclusion of the mediator, the intervention’s direct effects on follow-up outcomes were attenuated to varying degrees (Table 6). This attenuation pattern is consistent with, but does not establish, a possible role for SEC in the observed intervention-related differences. Overall, while the current three-wave design provides useful temporal ordering, the sample size and analytic scope preclude definitive causal or mechanistic conclusions. Accordingly, these mediation findings should be regarded as preliminary evidence broadly consistent with the proposed conceptual model and warrant replication in larger longitudinal trials.

### 3.5 Exploratory trajectory patterns and preliminary prognostic modeling

Building on the primary outcome analyses, this study further explored individual-level heterogeneity to examine why students may differ in the degree and maintenance of improvement following the intervention. Using longitudinal data from T0, T1, and T2, this section describes exploratory trajectory patterns in mental health indicators and presents a preliminary prognostic analysis.

**Figure 5:**
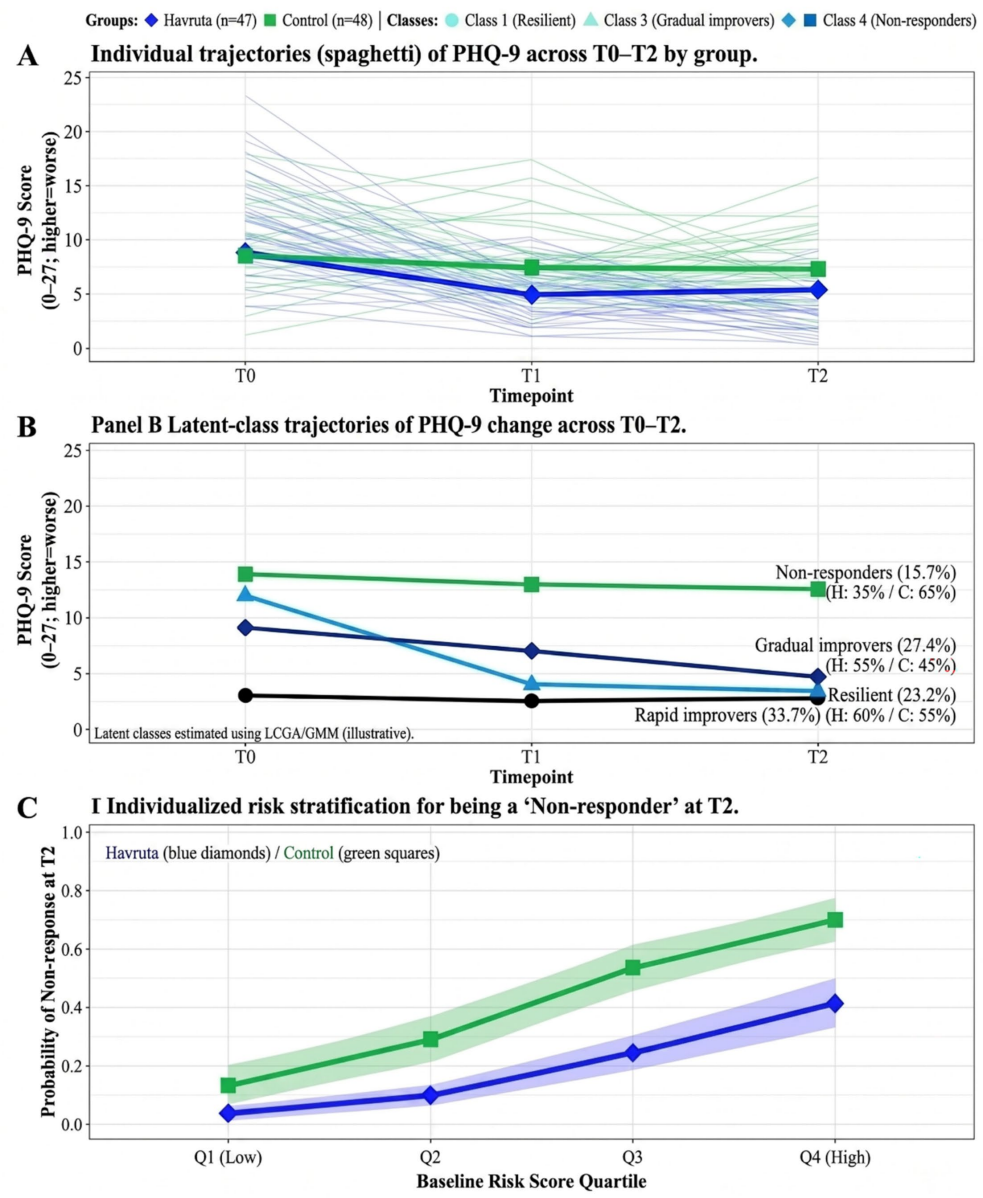
Exploratory individual and latent class trajectories of mental health across T0–T2 in the Havruta and control groups: **(A)** individual trajectories of PHQ-9 scores across T0–T2 by group, **(B)** latent-class trajectories of PHQ-9 change across T0–T2, and **(C)** modeled probability of being classified as a non-responder at T2 by baseline risk quartile.

In exploratory latent class growth analyses, the observed change patterns were most parsimoniously represented by four heterogeneous trajectory classes. The first class, termed Resilient, showed consistently low symptom levels across all time points. The second, Rapid improvers, showed marked symptom reduction immediately after the intervention that was maintained at follow-up. The third, Gradual improvers, demonstrated a delayed but continued reduction in symptoms over time. The fourth, Non-responders, showed persistently elevated symptoms with minimal longitudinal change. The proportion of improvement-related classes appeared higher in the intervention group, whereas the control group was more frequently represented in relatively stable or limited-improvement patterns. This distribution was directionally consistent with the overall intervention effect; however, given the exploratory nature of the analysis and the modest sample size, these subgroup patterns should be interpreted cautiously. The empirical distribution of these trajectories is presented in Figure 5. Specifically, Panel A shows individual spaghetti plots of depressive symptoms across time points in both groups. Panel B presents the estimated marginal means of the four latent classes. Panel C illustrates the modeled probability of being classified as a non-responder across baseline risk score quartiles in the intervention and control groups.

**Figure 6:**
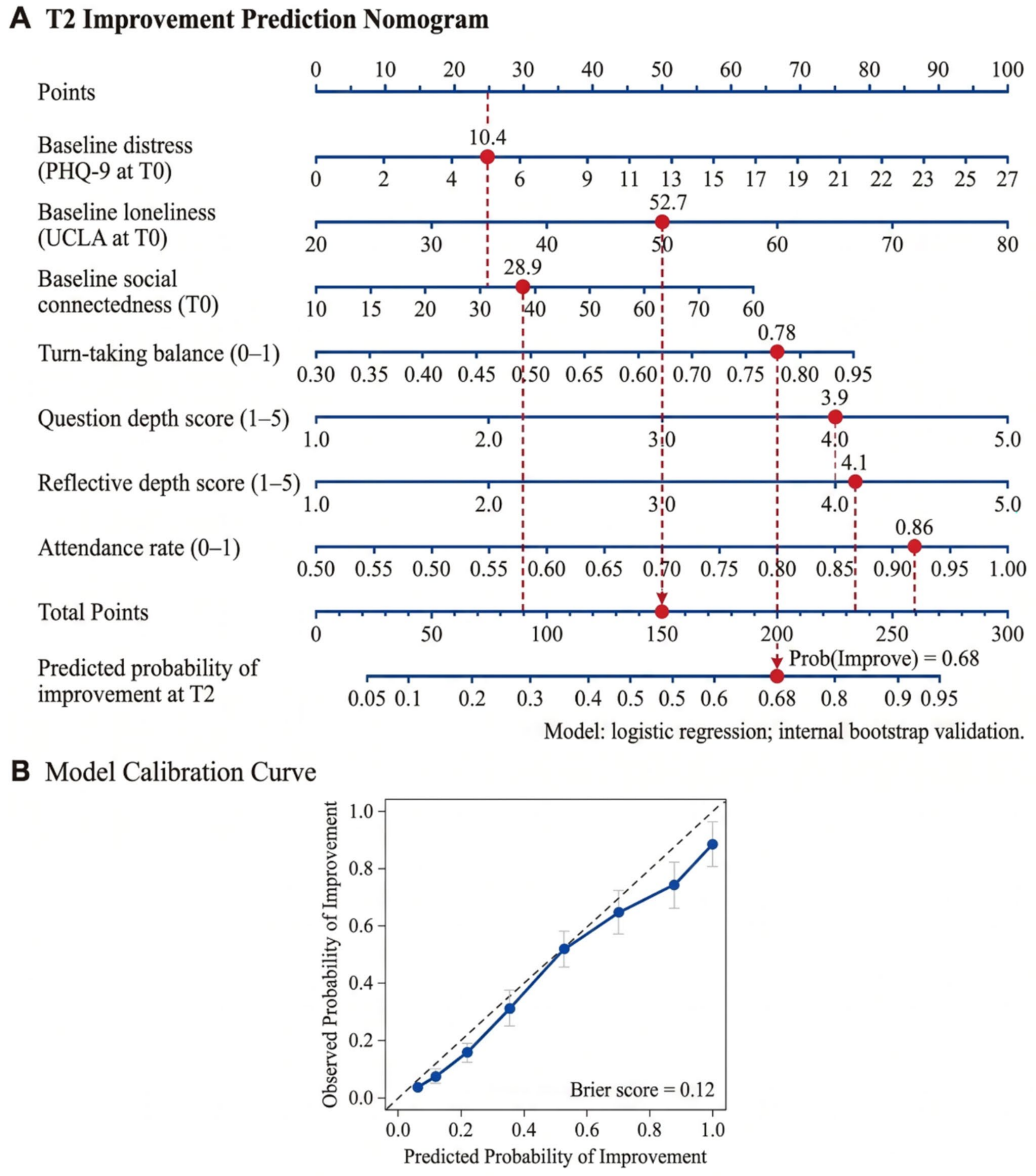
Exploratory nomogram for modeled probability of clinically meaningful improvement at follow-up based on baseline distress, loneliness, and dialogue-process indicators: **(A)** T2 improvement prediction nomogram and **(B)** model calibration curve.

For an initial hypothesis-generating examination of improvement probabilities, a multivariable logistic regression model incorporating baseline psychological distress, loneliness, and dialogue-process indicators was developed and visualized as an exploratory nomogram (Figure 6A). In this context, the nomogram provides a graphical representation of the fitted model, with each predictor scaled according to its relative statistical contribution; accordingly, only the predictor with the largest estimated effect reaches the 100-point maximum on the top axis. The red dots and vertical dashed lines in Panel A do not indicate distinct participant subgroups; instead, they illustrate a single hypothetical example showing how predictor values can be mapped onto the Points scale, summed as Total Points, and translated into a modeled probability of improvement.

Within this exploratory sample-specific model, higher baseline distress and loneliness were associated with higher modeled probabilities of clinically meaningful improvement, a pattern that may reflect greater room for change among students entering the trial with higher initial burden. Higher dialogue-process quality, including more balanced reciprocal speech, deeper questioning, and more stable attendance, was also associated with greater modeled probabilities of improvement. These associations should be interpreted as preliminary and hypothesis-generating rather than as validated prognostic determinants.

Figure 6B presents the calibration curve for the exploratory model, comparing the modeled improvement rates with the observed improvement rates in the current sample. The accompanying Brier score of 0.12 provides a descriptive summary of model performance in this dataset, with lower values indicating better probabilistic accuracy. However, in the absence of external validation, these findings should not be interpreted as evidence that the model is ready for applied screening, risk stratification, or routine use. Rather, they provide a preliminary framework for future prognostic research with larger, more diverse samples.

### 3.6 Multi-stakeholder triangulation and process evidence network

To obtain a broader understanding of intervention implementation beyond self-reported symptom change alone, this study triangulated self-reported outcomes with process evidence derived from peer ratings and implementer observations. Although these supplementary data do not constitute blinded outcome validation, they provide descriptive information on feasibility, participant engagement, and process features that may be relevant to interpreting the intervention context. This framework enabled a quantitative summary of implementation feasibility, engagement indicators, and perceived active elements. Overall, the findings suggested favorable acceptability, participant involvement, and contextual feasibility within this study setting. Broad convergence across informants was observed for the main descriptive patterns summarized in Table 7.

**Table 7:** Multi-stakeholder process evaluation: helpful components, engagement, feasibility, and perceived mechanisms of Havruta-structured dialogue.

Domain	Indicator/item	Student (*n* = 47)	Peer partner (*n* = 47)	Facilitator/RA
Engagement and dose	Sessions attended (0–8), mean (SD)	6.74 (1.12)		6.71 (1.09)
	Completion rate ≥6 sessions, n (%)	39 (83.0)		40 (84.8)
	Average in-session participation (1–5)	4.06 (0.61)	4.02 (0.64)	4.18 (0.55)
	Homework/reflection completion (0–8)	6.12 (1.49)		6.28 (1.36)
Feasibility and acceptability	Overall satisfaction (1–5)	4.28 (0.58)	4.21 (0.62)	
	Would recommend to classmates (1–5)	4.31 (0.63)	4.08 (0.71)	
	Perceived burden (1–5; higher = more burden)	2.08 (0.76)	2.14 (0.73)	
	Perceived psychological safety (1–5)	4.17 (0.59)	4.11 (0.63)	4.24 (0.52)
Most helpful components	Partner listening and being heard, n (%)	36 (76.6)	34 (72.3)	
	Asking “why/how” follow-up questions, n (%)	31 (66.0)	30 (63.8)	
	Summarizing/paraphrasing partner’s view, n (%)	29 (61.7)	28 (59.6)	
	Role switching (speaker/listener), n (%)	25 (53.2)	24 (51.1)	
	Joint meaning-making and reframing, n (%)	28 (59.6)	26 (55.3)	
Perceived mechanisms	Better emotion labeling/awareness (1–5)	4.03 (0.66)	3.96 (0.71)	
	Improved reappraisal/problem reframing (1–5)	3.92 (0.69)	3.88 (0.72)	
	Increased perspective-taking/empathy (1–5)	4.12 (0.62)	4.06 (0.65)	
	Stronger belonging/connectedness (1–5)	3.86 (0.74)	3.79 (0.77)	
	Reduced rumination (1–5)	3.71 (0.78)	3.63 (0.80)	
Implementation fidelity (observed)	Fidelity checklist score (0–10)			8.62 (0.93)
	Turn-taking balance (0–1)			0.78 (0.09)
	Question depth (1–5)			3.82 (0.58)
	Reflective depth (1–5)			3.94 (0.55)
	Protocol deviations, n (%) of sessions			5 (3.1)

Process metrics indicated that the intervention dosage and participant engagement were generally aligned, with adherence to the intended task structure overall acceptable. Participants reported relatively high satisfaction, a willingness to recommend the program, and comparatively low perceived burden. Taken together, these findings suggest that the dialogue structure was feasible and acceptable within the present campus context, although they should not be interpreted as evidence of readiness for broader implementation. Regarding perceived helpful elements, participants most frequently identified active listening, probing inquiry, structural paraphrasing, role reversal, and joint meaning construction. These perceptions are broadly consistent with the hypothesized process features of the Havruta protocol rather than providing direct confirmation of the underlying mechanisms. Observer ratings also suggested generally good implementation fidelity, and process indicators such as reciprocal participation, depth of questioning, and reflective depth were largely within the expected range. Collectively, these descriptive patterns support the interpretation that the intervention was delivered with reasonable consistency in this study.

Regarding perceived mechanisms of change, multi-stakeholder reports consistently highlighted improvements in emotional awareness, cognitive reappraisal, perspective-taking, and campus connectedness, alongside reductions in rumination. These qualitative insights were directionally consistent with the quantitative findings and provide one possible process-oriented interpretation of the observed outcome pattern. Building on this descriptive convergence, this study integrated conversational behaviors, SEC dimensions, and mental health outcomes to explore cross-domain coupling relationships.

**Figure 7:**
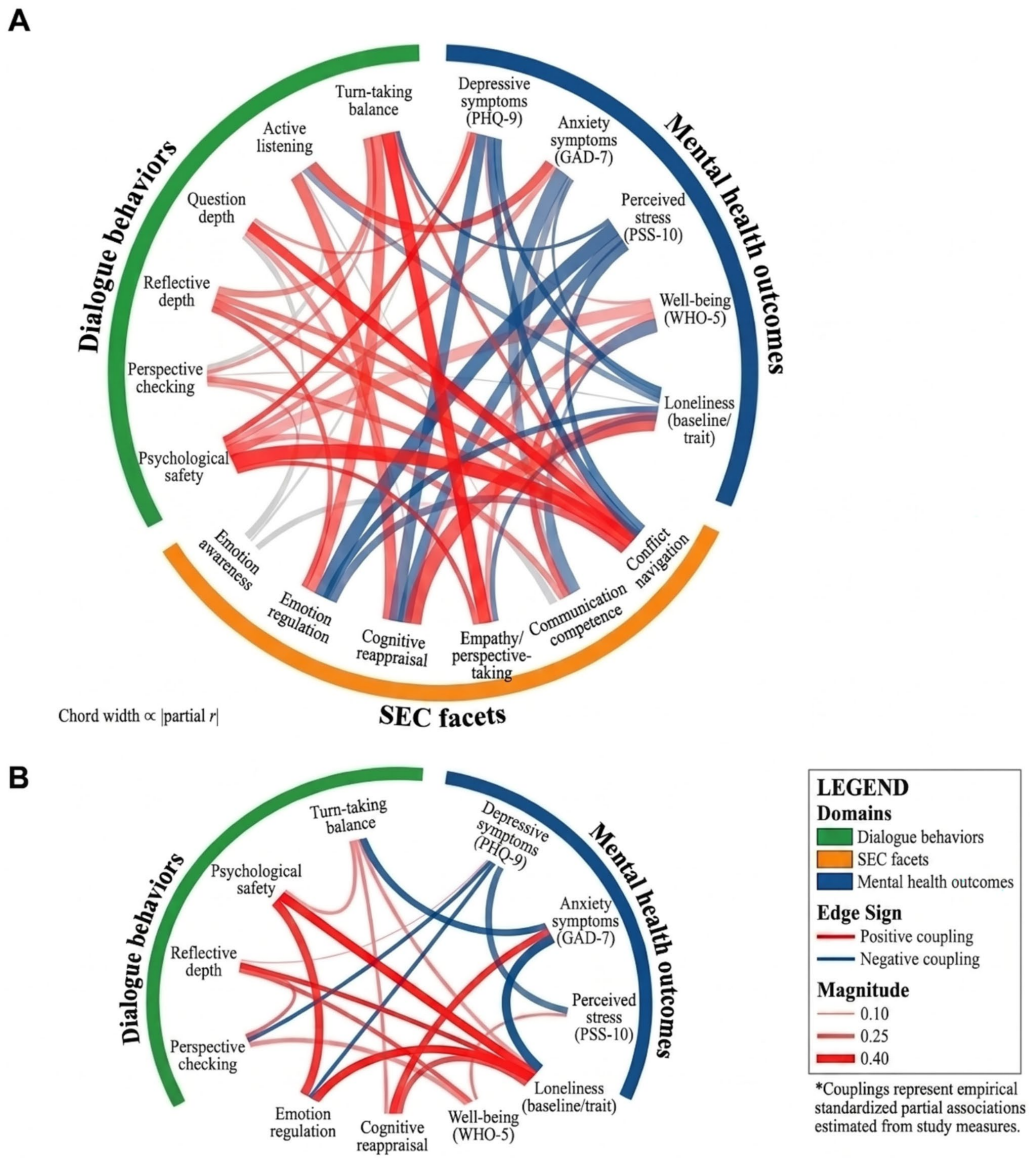
Chord diagram of cross-domain coupling between dialogue behaviors, social–emotional competence facets, and mental health outcomes: **(A)** full cross-domain coupling diagram and **(B)** simplified cross-domain coupling diagram.

The cross-domain coupling network analysis suggested closer associations among conversational process quality, SEC, and mental health indicators. In particular, stronger conversational process features appeared to be linked to more favorable SEC profiles, which, in turn, were associated with more favorable mental health outcomes. In contrast, the network also showed clustering among baseline risk-related nodes and symptom outcomes, together with relatively weaker connections to conversational quality and competence-related improvements. These structural patterns may offer one possible explanation for heterogeneity in responses, including the presence of students showing limited improvement. The cross-domain coupling relationships are displayed in Figure 7 as a chord diagram, in which edge weights and clustering patterns were derived from computed regularized partial correlations rather than from subjective interpretation.

## 4 Discussion

This study provides preliminary evidence that the Havruta-structured dialogue intervention may be a promising approach to campus mental health promotion in the current university context (Table 7). Compared with the control group, participants in the intervention condition demonstrated significantly greater and sustained reductions in depressive symptoms, anxiety, and perceived stress, alongside improved psychological wellbeing (Tables 2, 3). These findings are broadly consistent with prior literature suggesting that structured peer-mediated interventions may yield more durable psychological benefits than less structured forms of social support, providing external validation for the sustained improvements observed in our Havruta cohort (Ali et al., 2015; Pfeiffer et al., 2011; Repper and Carter, 2011; Wodika et al., 2025). Extending the principles outlined in previous studies (Balideh et al., 2025; Bavelas et al., 2000; Kuhn, 2015; Resnick et al., 2015; Weger et al., 2014), the structured interactional features of Havruta—such as active listening, reciprocal questioning, and role reversal—appear to uniquely provide conditions conducive to deeper emotional processing and self-regulation in interpersonal contexts (Balideh et al., 2025; Bavelas et al., 2000; Kuhn, 2015; Resnick et al., 2015; Weger et al., 2014).

From a mechanistic perspective, the present findings provide preliminary and exploratory evidence consistent with the possibility that enhancement of SEC may serve as an intermediary process linking the intervention to later mental health benefits (Table 6). The temporal association between improvement in SEC during the intervention phase and more favorable follow-up mental health outcomes is broadly consistent with theoretical models, established by previous research (ElBarazi, 2025; Kraag et al., 2006; Martins et al., 2010), that position emotional and social skill development as potentially important pathways of change. Our results provide novel empirical support for these models within a specifically structured dialogic framework. In parallel, dialogue processes such as probing, clarifying, and joint meaning construction (Figure 7) may support the development of competencies including cognitive reappraisal, perspective-taking, and empathic communication (Akbari et al., 2025). However, these interpretations should remain cautious. The mediation analyses do not establish a confirmed mechanism, and the observed pattern should be understood as preliminary evidence consistent with, rather than definitive proof of, the proposed conceptual model (Matthaeus et al., 2024).

Beyond the aggregate mean effects, the exploratory latent class analyses suggested four heterogeneous response trajectories—resilient, rapid improvers, gradual improvers, and non-responders (Figure 5)—highlighting the possibility that intervention response may vary meaningfully across individuals, a finding that aligns with the broader methodological consensus on treatment heterogeneity (Jung and Wickrama, 2008; Morin et al., 2011; Muthen and Muthen, 2000). This pattern is compatible with the broader argument that university mental health interventions may not operate uniformly across all students. In the exploratory moderation and prognostic analyses, participants with elevated baseline distress and lower social connectedness tended to show greater modeled probabilities of improvement. This pattern may reflect a possible compensatory benefit among students entering the intervention with a greater initial burden (Tables 4, 5). However, these subgroup patterns and modeled associations should be interpreted cautiously. The nomogram presented in Figure 6 should be regarded as an exploratory, hypothesis-generating framework rather than a pragmatic risk-stratification tool. Although combining baseline characteristics with dialogue-process indicators may offer a useful conceptual direction for future prognostic research, as recommended by recent methodological guidelines (Chen and Qi, 2025; Moons et al., 2015; Riley et al., 2013; Steyerberg et al., 2010), the present sample size is insufficient to support robust subgroup classification, applied screening, or individualized decision-making. Accordingly, these exploratory trajectory patterns and preliminary predictive models require replication and external validation in substantially larger and more diverse cohorts before any broader practical use can be considered.

With respect to translational relevance, the multi-stakeholder evidence supports the feasibility and acceptability of the Havruta protocol within the present campus setting (Table 7) (Durlak and DuPre, 2008; Proctor et al., 2011). At the same time, several limitations should temper interpretation. This study relied on a modest convenience sample, and students experiencing acute psychological crises were explicitly excluded, limiting the generalizability of the findings (Di Consiglio et al., 2022). For this reason, this study is best understood as providing preliminary evidence of efficacy and contextual feasibility rather than demonstrating broad effectiveness or readiness for immediate scale-up. Future research should therefore evaluate this dialogic framework in fully powered, multi-center trials with longer follow-up periods, while also testing whether the exploratory secondary findings reported here—including the mediation, trajectory, and prognostic analyses—can be replicated. Such work would also be necessary to clarify which students may require augmented or alternative forms of support when standard Havruta training yields limited benefit.

## 5 Conclusion

This study examined the effects of a Havruta-structured dialogue intervention on the mental health of university students. Compared with an active control condition, participants in the intervention group showed significant and sustained reductions in depressive symptoms, anxiety, and perceived stress, together with improvements in psychological wellbeing and SEC. Exploratory mediation analyses yielded preliminary evidence consistent with SEC as one plausible intermediary associated with these favorable mental health outcomes, although the present design does not allow definitive mechanistic conclusions. In addition, exploratory trajectory and prognostic analyses suggested heterogeneous response patterns, with baseline psychological distress, loneliness, and dialogue-process indicators emerging as candidate correlates of differential improvement rather than confirmed predictors of benefit. Multi-stakeholder evaluations further supported the intervention’s feasibility and acceptability within the present campus context. Overall, these findings indicate promising preliminary effects, but broader effectiveness, generalizability, and applied prognostic utility remain to be established. Future research should prioritize replicating these findings in larger and more diverse samples, externally validating the exploratory secondary analyses, and refining intervention strategies for students who may respond less well to standard dialogic training.

## Data Availability

The raw data supporting the conclusions of this article will be made available by the authors, without undue reservation.
